# Prediction of Alzheimer’s Disease-Associated Genes by Integration of GWAS Summary Data and Expression Data

**DOI:** 10.3389/fgene.2018.00653

**Published:** 2019-01-07

**Authors:** Sicheng Hao, Rui Wang, Yu Zhang, Hui Zhan

**Affiliations:** ^1^College of Computer and Information Science, Northeastern University, Boston, MA, United States; ^2^Department of Neurosurgery, Heilongjiang Province Land Reclamation Headquarters General Hospital, Harbin, China; ^3^College of Electronic Engineering, Heilongjiang University, Harbin, China

**Keywords:** Alzheimer’s disease, genome-wide association study, autoimmune diseases, transcriptome-wide association study, false discover rate

## Abstract

Alzheimer’s disease (AD) is the most common cause of dementia. It is the fifth leading cause of death among elderly people. With high genetic heritability (79%), finding the disease’s causal genes is a crucial step in finding a treatment for AD. Following the International Genomics of Alzheimer’s Project (IGAP), many disease-associated genes have been identified; however, we do not have enough knowledge about how those disease-associated genes affect gene expression and disease-related pathways. We integrated GWAS summary data from IGAP and five different expression-level data by using the transcriptome-wide association study method and identified 15 disease-causal genes under strict multiple testing (α < 0.05), and four genes are newly identified. We identified an additional 29 potential disease-causal genes under a false discovery rate (α < 0.05), and 21 of them are newly identified. Many genes we identified are also associated with an autoimmune disorder.

## Introduction

Alzheimer’s disease (AD) is the most common cause of dementia which is characterized by a decline in cognitive skills that affects a person’s ability to perform everyday activities. Estimated 5.4 million people in the United States are living with AD. It is the fifth-leading cause of death among those age 65 and older (Alzheimer’s Association, 2016). Although some drugs showing effectiveness to mitigate the symptoms from getting worse for a limit time, no treatment can stop the disease. Heritability for the AD was estimated up to 79% (Gatz et al., 2006). However, the current finding of AD-associated genetic variants is not enough to fully explain the AD signal pathway in sufficient detail.

During recent years, with the rapid advance of next-generation DNA sequencing, identify disease-related mutation from large data set and develop treatment become possible (Cheng et al., 2016a, 2018a,b). Genome-wide comparison studies (GWASs) have identified a significant amount of common genetic variants associated with complex traits and diseases (Welter et al., 2014; Hu et al., 2017a,b). Many previous studies have identified genes such as APOE (Mahoney-Sanchez et al., 2016; Liao et al., 2017) on chromosome 19. However, the causal relation of those associated genes and variants remain unclear. For example, recent study and data showed that a female with the APOE gene under greater risk than a male with the APOE gene (Cacciottolo et al., 2016; Mazure and Swendsen, 2017). This strongly indicates that we have little knowledge about how this risk factor effect people.

With GWAS summary data provided by the International Genomics of Alzheimer’s Project (IGAP) (Lambert et al., 2013), we are able to study AD in great detail. For a complex disease such as AD, the top single nucleotide polymorphisms (SNPs) often located in the non-coding region, hard to know which gene is modified by that mutation and many significant SNPs are in high linkage disequilibrium (LD) with non-significant SNPs, plus many associated SNPs are more likely to locate in expression regulation region of the disease causal gene (Nicolae et al., 2010; Karch et al., 2016). To identify disease-associated genes, we used the transcriptome-wide association study (TWAS) (Gusev et al., 2016) method which integrates GWAS summarization level data, expression level data from human tissue. TWAS method can eliminate potential confounding and find disease causal gene by focusing only on expression trait linking related by genetic variation; it can also increase statistical power from the lower multiple-testing burden and the noise reduction of gene expression from environmental factors (Gusev et al., 2016).

Previous studies have pointed out at AD is closely related to autoimmune disorders (D’Andrea, 2005; Carter, 2010). After detecting possible disease causal gene for AD, we manually curated existing research about the autoimmune diseases that potentially related to AD.

## Materials and Methods

Data we used for SNP-trait association is a large-scale GWAS summary data provided by IGAP with total 17,008 AD cases and 37,154 controls, include 7,055,881 SNPs, we selected 6,004,159 SNPs. Expression level data are from adipose tissue (RNA-seq), whole blood (RNA array), peripheral blood (RNA array), brain tissue (RNA-seq and RNA-seq splicing) (Raitakari et al., 2008; Nuotio et al., 2014; Wright et al., 2014; Fromer et al., 2016). Selection method can be find in Supplementary Materials.

### Transcriptome-Wide Association Study

Transcriptome-wide association study can be viewed as a test for correlation between predicted gene expression and traits from GWAS summary association data. The predicted effect size of gene expression on traits can be viewed as a linear model of genotypes with weights based on the correlation between SNPs and gene expression in the training data while accounting for LD among SNPs.

**FIGURE 1:**
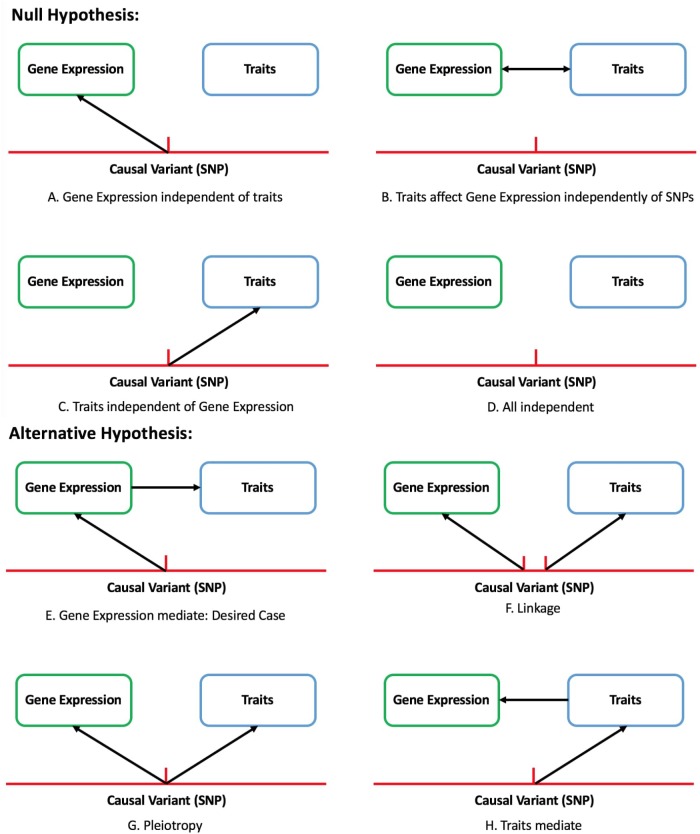
Eight of causal assumption between gene, expression and trait in TWAS study. Null hypothesis: gene expression is completely independent of traits **(A–D)**. Alternative hypothesis: causal relation exists between SNPs and traits **(E–H)**.

There are eight modes of causality for the relationship between genetic variant, gene expression, and traits. Scenarios Figures 1E–H should be identified as significant by TWAS and its corresponding null hypothesis is gene expression completely independent of traits (Figures 1A–D). By only focusing on the genetic component of expression, the instances of expression-trait association that is not caused by genetic variation but variation in traits can be avoided. One aspect that needs to be noticed is, same as other methods, TWAS is also confounded by linkage and pleiotropy.

### Performing TWAS With GWAS Summary Statistics

We integrated gene expression measurements from five tissues with summary GWAS to perform multi-tissue transcriptome-wide association. In each tissue, TWAS used cross-validation to compare predictions from the best *cis*-eQTL to those from all SNPs at the locus. Prediction models choosing from BLUP (Lofgren et al., 1989), BSLLM (Zhou et al., 2013), LASSO (Tibshirani, 1997), and elastic net (Gamazon et al., 2015).

Transcriptome-wide association study Imputes effect size (z-score) of the expression and trait are linear combination of elements of z-score of SNPs for traits with weights. The weights, W = ∑ _e,s_ $\sum {}_{\text{s,s}}^{-1}$, are calculated using ImpG-Summary algorithm (Pasaniuc et al., 2014) and adjusted for LD. ∑ _e,s_ is the estimated covariance matrix between all SNPs at the locus and gene expression and ∑ _s,s_ is the estimated covariance among all SNPs which is used to account for LD.

Standardized effect sizes (Z-scores) of SNPs for a trait at a given cis locus can be denoted as a vector *Z*. Also, the imputed Z-score of expression and trait, *WZ*, has variance. *W* ∑_s,s_*W*^t^. Therefore, the imputation *Z* score of the *cis* genetic effect on the trait is,

$$Z_{\text{TWAS}}=\mathrm{WZ}/{\left(W\Sigma_{\text{s,s}}W^{\text{t}}\right)}^{\frac{1}{2}}.$$

Bonferroni correction is usually applied when identifying significant disease-associated gene. The standard multiple testing conducted in TWAS is 0.05/15000 (Gusev et al., 2016). But traditional *p*-value cutoffs adjusted by Bonferroni correction are made too strict in order to avoid an abundance of false positive results. The thresholds like 0.05/15000 for significant genes are usually chosen so that the probability of any single false positive among all loci tested is smaller than 0.05, which will lead to many missed findings. Instead, False Discovery Rate error measure is a more useful approach when a study involves a large number of tests, since it can identify as many significant genes as possible while incurring a relatively low proportion of false positives (Storey and Tibshirani, 2003). For each tissue, we used the Benjamini and Hochberg procedure (Benjamini and Hochberg, 1995) in addition to the Bonferroni correction for all gene tested. The Benjamini and Hochberg procedure is one of false discovery rate procedures that are designed to control the expected proportion of false positives. It is less stringent than the Bonferroni correction, thus has greater power. Since this is study is more exploratory, we can pay more risk of type I error for larger statistical power. It works as follows:

Put individual *p*-values in ascending order and assign ranks to the *p*-values.

(1) Calculate each individual Benjamini and Hochberg critical value with formula $\frac{k}{m}$α, where *k* is individual *p*-value’s rank, *m* is total number of tests and α is the false discovery rate.
(2) Find the largest *k* such that *P*_k_ ≤$\frac{k}{m}$α and reject the null hypothesis for all *H*_i_ for *i* = 1, ...*k*.

## Results

To determine which gene is significantly associated with AD, we first performed strict multiple testing Bonferroni correction (*p*-value < 0.05/15000). We found 15 significant genes (Table 1), 11 of them has identified by previous studies of AD. In order to increase the search range, we performed false discovery rate under the same alpha (0.05). After the Benjamini and Hochberg procedure (Benjamini and Hochberg, 1995), we found 29 additional genes (Table 2). Nine of those genes has previously identified to be related to AD.

**Table 1:** Significant genes identified by TWAS under strict multiple testing.

Gene	Chromosome	Tissue	*P*-Value	Z-score	Related to autoimmune diseases
PVRL2	19	Brain (CMC) RNA-seq	4.92E-34	−12.1626	Yes
TOMM40	19	Whole Blood (YFS) RNA Arr ay	1.13E-25	10.4749	
CLPTM1	19	Brain (CMC) RNA-seq	5.73E-17	−8.37061	
CLU	8	Brain (CMC) RNA-seq splicing	1.45E-16	−8.26075	
CR1	1	Brain (CMC) RNA-seq	4.08E-15	7.8523	Yes
CEACAM19	19	Adipose (METSIM) RNA-seq	3.38E-11	6.62905	Yes
MS4A6A	11	Whole Blood (YFS) RNA Array	2.92E-10	6.30316	
TRPC4AP	20	Brain (CMC) RNA-seq splicing	9.43E-10	6.1188	
MLH3	14	Brain (CMC) RNA-seq splicing	7.86E-09	−5.77148	Yes
MS4A6A	11	Peripheral Blood (NTR) RNA Array	5.72E-08	5.4272	
PTK2B	8	Peripheral Blood (NTR) RNA Array	9.93E-08	5.32809	
PVR	19	Brain (CMC) RNA-seq	2.05E-07	−5.19443	Yes
PICALM	11	Peripheral Blood (NTR) RNA Array	2.84E-07	5.1337	Yes
MS4A4A	11	Adipose (METSIM) RNA-seq	6.11E-07	4.99	
BIN1	2	Whole Blood (YFS) RNA Array	1.18E-06	4.859114	
FNBP4	11	Whole Blood (YFS) RNA Array	1.49E-06	−4.81307	
PTK2B	8	Whole Blood (YFS) RNA Array	2.89E-06	4.6784	Yes
BIN1	2	Peripheral Blood (NTR) RNA Array	3.24E-06	4.65503	Yes

**Table 2:** Additional gene under Benjamini and Hochberg procedure.

Gene	Chromosome	Tissue	*P*-Value	Z-score	Previously identified
PHACTR1	6	Whole Blood (YFS) RNA Array	3.41E-06	−4.64434	
PTPMT1	11	Whole Blood (YFS) RNA Array	4.45E-06	4.58895	
MTCH2	11	Peripheral Blood (NTR) RNA Array	5.76E-06	4.535	
C1QTNF4	11	Adipose (METSIM) RNA-seq	8.82E-06	4.44	
FAM180B	11	Brain (CMC) RNA-seq	1.09E-05	−4.39814	Yes
DMWD	19	Whole Blood (YFS) RNA Array	1.22E-05	4.3733	
ELL	19	Whole Blood (YFS) RNA Array	1.89E-05	4.277	Yes
ZNF740	12	Brain (CMC) RNA-seq splicing	2.08E-05	4.25599	
NYAP1	7	Adipose (METSIM) RNA-seq	2.47E-05	−4.21777	
SDAD1	4	Whole Blood (YFS) RNA Array	3.04E-05	−4.17062	
MTSS1L	16	Brain (CMC) RNA-seq splicing	3.35E-05	4.14833	
PHKB	16	Brain (CMC) RNA-seq	3.70E-05	−4.1257	Yes
SLC39A13	11	Brain (CMC) RNA-seq splicing	4.01E-05	−4.10667	Yes
CD33	19	Whole Blood (YFS) RNA Array	4.04E-05	4.1051	Yes
AP2A2	11	Brain (CMC) RNA-seq	4.28E-05	−4.09193	Yes
ZYX	7	Adipose (METSIM) RNA-seq	4.56E-05	−4.07718	
ZNF232	17	Brain (CMC) RNA-seq splicing	4.73E-05	−4.0688	
ZNF232	17	Brain (CMC) RNA-seq splicing	4.76E-05	4.0671	
DLST	14	Peripheral Blood (NTR) RNA Array	5.26E-05	4.0436	Yes
TBC1D7	6	Adipose (METSIM) RNA-seq	5.34E-05	4.0403	
ELL	19	Adipose (METSIM) RNA-seq	5.48E-05	4.03401	
SLC39A13	11	Brain (CMC) RNA-seq splicing	5.79E-05	−4.02128	Yes
TMCO6	5	Whole Blood (YFS) RNA Array	6.50E-05	3.9938	
CEL	9	Whole Blood (YFS) RNA Array	6.99E-05	3.97671	Yes
MYBPC3	11	Adipose (METSIM) RNA-seq	7.05E-05	3.97	Yes
TBC1D7	6	Brain (CMC) RNA-seq splicing	7.48E-05	−3.96063	
LRRC25	19	Peripheral Blood (NTR) RNA Array	7.74E-05	−3.9523	
TBC1D7	6	Brain (CMC) RNA-seq splicing	8.37E-05	3.93351	
KIR3DX1	19	Peripheral Blood (NTR) RNA Array	8.87E-05	3.9195	
SIX5	19	Peripheral Blood (NTR) RNA Array	9.32E-05	3.9076	
HBEGF	5	Whole Blood (YFS) RNA Array	9.92E-05	−3.8926	Yes
NUP88	17	Peripheral Blood (NTR) RNA Array	1.60E-04	−3.7748	
FAM105B	5	Whole Blood (YFS) RNA Array	1.61E-04	3.773	
ARL6IP4	12	Peripheral Blood (NTR) RNA Array	2.10E-04	3.707	

**Gene**	**Chromosome**	**Tissue**	***P*-Value**	**Z-score**	
	
PHACTR1	6	Whole Blood (YFS) RNA Array	3.41E-06	−4.64434	
PTPMT1	11	Whole Blood (YFS) RNA Array	4.45E-06	4.58895	
MTCH2	11	Peripheral Blood (NTR) RNA Array	5.76E-06	4.535	
C1QTNF4	11	Adipose (METSIM) RNA-seq	8.82E-06	4.44	
FAM180B	11	Brain (CMC) RNA-seq	1.09E-05	−4.39814	
DMWD	19	Whole Blood (YFS) RNA Array	1.22E-05	4.3733	
ELL	19	Whole Blood (YFS) RNA Array	1.89E-05	4.277	
ZNF740	12	Brain (CMC) RNA-seq splicing	2.08E-05	4.25599	
NYAP1	7	Adipose (METSIM) RNA-seq	2.47E-05	−4.21777	
SDAD1	4	Whole Blood (YFS) RNA Array	3.04E-05	−4.17062	
MTSS1L	16	Brain (CMC) RNA-seq splicing	3.35E-05	4.14833	
PHKB	16	Brain (CMC) RNA-seq	3.70E-05	−4.1257	
SLC39A13	11	Brain (CMC) RNA-seq splicing	4.01E-05	−4.10667	
CD33	19	Whole Blood (YFS) RNA Array	4.04E-05	4.1051	
AP2A2	11	Brain (CMC) RNA-seq	4.28E-05	−4.09193	
ZYX	7	Adipose (METSIM) RNA-seq	4.56E-05	−4.07718	
ZNF232	17	Brain (CMC) RNA-seq splicing	4.73E-05	−4.0688	
ZNF232	17	Brain (CMC) RNA-seq splicing	4.76E-05	4.0671	
DLST	14	Peripheral Blood (NTR) RNA Array	5.26E-05	4.0436	
TBC1D7	6	Adipose (METSIM) RNA-seq	5.34E-05	4.0403	
ELL	19	Adipose (METSIM) RNA-seq	5.48E-05	4.03401	
SLC39A13	11	Brain (CMC) RNA-seq splicing	5.79E-05	−4.02128	
TMCO6	5	Whole Blood (YFS) RNA Array	6.50E-05	3.9938	
CEL	9	Whole Blood (YFS) RNA Array	6.99E-05	3.97671	
MYBPC3	11	Adipose (METSIM) RNA-seq	7.05E-05	3.97	
TBC1D7	6	Brain (CMC) RNA-seq splicing	7.48E-05	−3.96063	
LRRC25	19	Peripheral Blood (NTR) RNA Array	7.74E-05	−3.9523	
TBC1D7	6	Brain (CMC) RNA-seq splicing	8.37E-05	3.93351	
KIR3DX1	19	Peripheral Blood (NTR) RNA Array	8.87E-05	3.9195	
SIX5	19	Peripheral Blood (NTR) RNA Array	9.32E-05	3.9076	
HBEGF	5	Whole Blood (YFS) RNA Array	9.92E-05	−3.8926	
NUP88	17	Peripheral Blood (NTR) RNA Array	1.60E-04	−3.7748	
FAM105B	5	Whole Blood (YFS) RNA Array	1.61E-04	3.773	
ARL6IP4	12	Peripheral Blood (NTR) RNA Array	2.10E-04	3.707	

PVRL2 (*p*-value 4.92^∗^10ˆ–34 in Brain (CMC) RNA-seq, also known as NECTIN2) is a well-known gene for AD. This gene encodes a single-pass type I membrane glycoprotein and interact with AOPE gene (Kulminski et al., 2018). TOMM40 [*p*-value 1.13^∗^10ˆ–25 in Whole Blood (YFS) RNA Array] is also located adjacent to APOE. It has been identified by previous studies worldwide as AD related gene (Lyall et al., 2014; Goh et al., 2015; Mise et al., 2017). It is the central and essential component of the translocase of the outer mitochondrial membrane (Humphries et al., 2005). This confirmed that mitochondrial dysfunction plays a significant role in AD-related pathology (Swerdlow and Khan, 2004; Roses et al., 2016).

Other highly connected genes function group identified are BIN1 [*p*-value 1.18 × 10^−6^in Whole Blood (YFS) RNA Array; 3.24 × 10^−6^ in Peripheral Blood (NTR) RNA Array], CLU (*p*-value 1.45 × 10^−16^), MS4A6A [*p*-value 5.72 × 10^−8^in Peripheral Blood (NTR) RNA Array; 2.92 × 10^−10^in Whole Blood (YFS) RNA Array] (Han et al., 2017).

### New Identified Genes

MLH3 [*p*-value 7.86 × 10^−9^ in Brain (CMC) RNA-seq splicing] FNBP4 [*p*-value 1.49 × 10^−6^in Whole Blood (YFS) RNA Array], CEACAM19 [*p*-value 3.38 × 10^−11^ in Adipose (METSIM) RNA-seq], and CLPTM1 [*p*-value 5.73 × 10^−17^ in Brain (CMC) RNA-seq] are newly identified AD-associated genes. MLH3 gene is known for its function in repair mismatched DNA and risk for thyroid cancer and lupus (Souliotis et al., 2016; Al-Sweel et al., 2017; Javid et al., 2018). CEACAM19 gene located in chromosome 19, a previous study showed high expression of CEACAM19 for patients with breast cancer (Estiar et al., 2017); CLPTM1 has been shown to increase the risk of lung cancer and melanoma (Llorca-Cardenosa et al., 2014; Lee et al., 2017). Both CEACAM19 and CLPTM1 gene are located in chromosome 19 and near APOE gene. More detailed studies are needed to investigate the relationship between those genes and whether CLPTM1 and CEACAM19 are disease causal gene.

## Discussion

### APOE Related Genes

**FIGURE 2:**
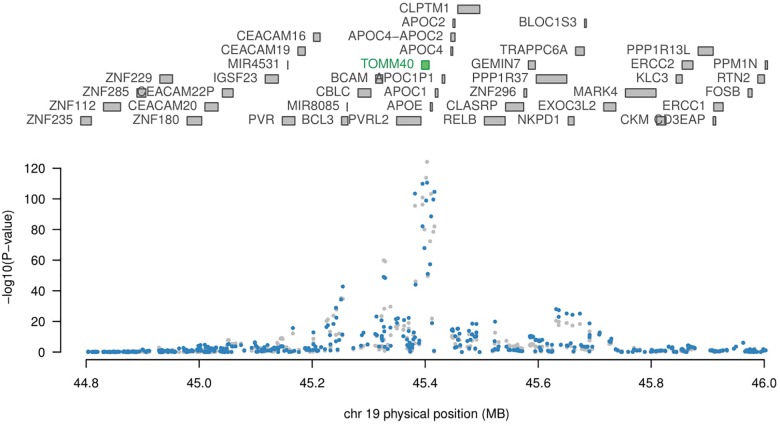
Geneposition plot in chromosome 19. Expression data: whole blood.

Although APOE is not reported to be significant in any tissue, not enough evidence to conclude that APOE is not related to AD. Since each SNP has a weight assigned regarding the expression in TWAS study, even two genes are both significantly related to a disease, it is very likely only one of them will be showing significant in TWAS. TOMM40 (Figure 2, *p*-value 1.13 × 10^−25^) gene located adjacent to APOE (Pomara et al., 2011), and has a strong LD with APOE gene (Yu et al., 2007), hence TWAS didn’t detect this APOE does not imply it is not disease causal gene. APOE and TOMM40 may interact to affect AD pathology such as mitochondrial dysfunction (David et al., 2005; Roses et al., 2013). Further study is needed to show causal relation in detail. PICALM [*p*-value 2084 × 10^−7^ in Peripheral Blood (NTR) RNA Array] and PTK2B [*p*-value 9.93 × 10^−8^ in Peripheral Blood (NTR) RNA Array; *p*-value 2.89 × 10^−6^ in Whole Blood (YFS) RNA Array] are also related to APOE and TOMM40 gene according to previous studies (Carter, 2011; Gharesouran et al., 2014; Morgen et al., 2014; Han et al., 2017).

### Association With Autoimmune Diseases

Complex disease such as AD, often shares common pathways or causal genes with other diseases (Hu et al., 2017c). For instance, TOMM40 is a shared disease-associated gene between AD and Type II diabetes (Greenbaum et al., 2014). Recent studies showing autoimmune diseases have closed relation with AD (D’Andrea, 2005; Lehrer and Rheinstein, 2015; Wotton and Goldacre, 2017). Among all the genes we identified through TWAS method, eight of them are related to autoimmune diseases.

**FIGURE 3:**
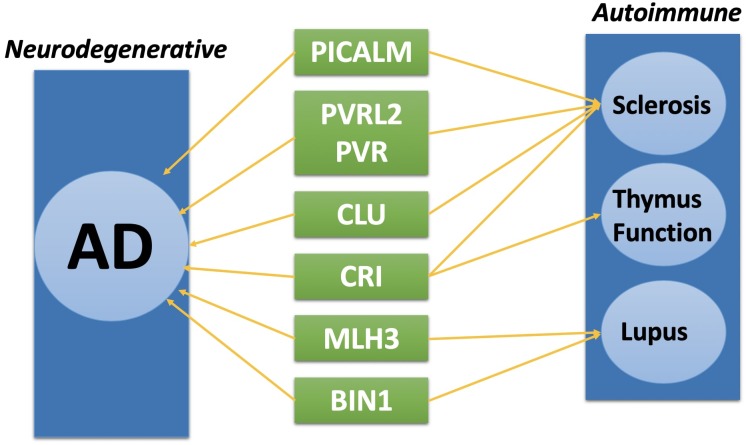
Shared disease associated gene between Alzheimer’s disease (AD) and Autoimmune diseases.

As shown in Figure 3, PICALM, PVRL2, PVR, and CLU have shown to be related to systemic, an autoimmune disease characterized by vascular injury and debilitating tissue fibrosis (Xia et al., 2010; Ryu et al., 2014; Tsou et al., 2016; van Luijn et al., 2016). CR1 and CLU gene are related to thymus function which could potentially cause an autoimmune disorder (French et al., 1992; Pekalski et al., 2017). MLH3 and BIN1 gene have shown to be associated with Lupus, another severe autoimmune disease (Armstrong et al., 2014; Souliotis et al., 2016). Although with existing result, we don’t have enough evidence to prove these genes are both disease causal genes for AD and autoimmune disease, further research from areas such as metabolomics and proteomics is needed to study the disease association between AD and autoimmune diseases (Cheng et al., 2016b, 2017; Hu et al., 2018).

## Author Contributions

RW and YZ wrote the method manuscript. SH and HZ analyzed the data and wrote the manuscript. All authors read and approved the final manuscript.

## Conflict of Interest Statement

The authors declare that the research was conducted in the absence of any commercial or financial relationships that could be construed as a potential conflict of interest.
