# Supplementary material for: Prediction of Alzheimer’s Disease-Associated Genes by Integration of GWAS Summary Data and Expression Data
Source: Front Genet. 2019 Jan 7;9:653. doi: 10.3389/fgene.2018.00653 (PMC6330278; doi:10.3389/fgene.2018.00653)
Supplement: Supplementary file 1 [file Table_1.DOCX]

# Statement of reproducibility

This is the code for research article Prediction of Alzheimer's disease-associated genes by integration of GWAS summary data and expression data.

<https://github.com/sichenghao1992/TWAS_AD>

Each part of the code is independent from another.
